# Probabilistic Inference of Biological Networks via Data Integration

**DOI:** 10.1155/2015/707453

**Published:** 2015-03-22

**Authors:** Mark F. Rogers, Colin Campbell, Yiming Ying

**Affiliations:** ^1^Intelligent Systems Laboratory, University of Bristol, Merchant Venturers Building, Bristol BS8 1UB, UK; ^2^College of Engineering, Mathematics and Physical Sciences, University of Exeter, Harrison Building, North Park Road, Exeter EX4 4QF, UK

## Abstract

There is significant interest in inferring the structure of subcellular networks of interaction. Here we consider supervised interactive network inference in which a reference set of known network links and nonlinks is used to train a classifier for predicting new links. Many types of data are relevant to inferring functional links between genes, motivating the use of data integration. We use pairwise kernels to predict novel links, along with multiple kernel learning to integrate distinct sources of data into a decision function. We evaluate various pairwise kernels to establish which are most informative and compare individual kernel accuracies with accuracies for weighted combinations. By associating a probability measure with classifier predictions, we enable cautious classification, which can increase accuracy by restricting predictions to high-confidence instances, and data cleaning that can mitigate the influence of mislabeled training instances. Although one pairwise kernel (the tensor product pairwise kernel) appears to work best, different kernels may contribute complimentary information about interactions: experiments in *S. cerevisiae* (yeast) reveal that a weighted combination of pairwise kernels applied to different types of data yields the highest predictive accuracy. Combined with cautious classification and data cleaning, we can achieve predictive accuracies of up to 99.6%.

## 1. Introduction

There is a significant interest in determining subcellular network structures, from metabolic and protein-protein interaction networks, through to signalling pathways. Two broad interactive inference approaches are unsupervised and supervised network inference. With unsupervised inference, no prior knowledge of network linkage is assumed. Supervised inference is a more tractable alternative in which there is a training set of links and nonlinks, believed to be reliably known, and the task is to train a classifier using this information. We then make predictions for additional possible links where interactive network structure is less clearly resolved. One advantage of supervised inference is that there are a variety of pathways where the structure is fairly reliably determined and thus this prior structural knowledge could give a viable training set. A further advantage of supervised inference is that different types of data are informative about whether a functional link may exist, allowing practitioners to integrate data from diverse sources [1]. Furthermore we can weight these different data sources according to their relative significance. With unsupervised learning, it is much more difficult integrating different types of data into a predictive model, though various schemes have been suggested.

In this paper we will consider supervised network inference and we evaluate a variety of strategies to improve predictive performance. First we consider multiple kernel learning (MKL) in which different types of data are encoded into different pairwise base kernels. Using a weighted combination of base kernels, we construct a composite kernel that is used in a kernel-based classifier, for example, a Support Vector Machine (SVM) [2]. In Section 3 we show that this integrative approach gives better performance over a uniform weighting of kernels or classifiers constructed using only one type of data. Secondly, we discuss both established and a novel pairwise kernel for use with MKL. In this study we are interested in functional links between pairs of nodes in an interactive network, so the kernels we use encode similarity between pairs. Our goal is to investigate which pairwise kernel is best and whether a variety of such pairwise kernels should be used in combination with MKL. Next we associate a probability measure with the predicted class assignment. This facilitates cautious classification and motivates a novel data cleaning method. We demonstrate dramatic improvements in accuracy via cautious classification, in which test accuracy is improved at the expense of making predictions for only a subset of possible links or nonlinks. This probability measure also motivates a method for data cleaning: we train a classifier incrementally and predict a new link-label prior to adding it to our training set. If, with a high confidence prediction, the predicted link-label disagrees with the actual label then this may indicate an outlier (a wrong link-label) and the datapoint should not be learnt. We investigate a method of incremental data cleaning for SVMs in which we sequentially add training data to the training set by selecting the next example closest to the current separating hyperplane: these are necessarily low confidence predictions and, by this means, we defer encounter with potential outliers toward the end of the sequential learning process. For the data set considered we show that this strategy leads to a small improvement in test accuracy.

## 2. Methods

### 2.1. Pairwise Kernels

*Kernels* [2, 3] encode the similarity of data objects and they can be constructed for a variety of different types of data, from continuously valued to sequence or graph information [2, 4]. For network inference, we will use a label *y*_*i*_1_,*i*_2__ = +1 for a functional interaction between a pair of nodes (e.g., genes), labelled *i*_1_ and *i*_2_. *y*_*i*_1_,*i*_2__ = −1 will label a noninteracting pair. Thus, with supervised inference, we have an adjacency matrix with components +1 and −1 and a number of unknown elements which we wish to estimate.

Our data is in the form **x**_*i*_ (where *i* = 1,…, *m*). Linkage patterns in the data are classified in terms of pairings of nodes and appropriate kernels quantify a similarity between pairs. Thus, a comparison between a pair (**x**_*i*_1__, **x**_*i*_2__) and a further pair (**x**_*i*_3__, **x**_*i*_4__) could be performed through a comparison of **x**_*i*_1__ with **x**_*i*_3__ and **x**_*i*_2__ with **x**_*i*_4__ and, secondly, **x**_*i*_1__ with **x**_*i*_4__ and **x**_*i*_2__ with **x**_*i*_3__. If we write a general pairwise kernel as ${\hat{K}}_{P}={\hat{K}}_{P}((x_{i_{1}},x_{i_{2}}),(x_{i_{3}},x_{i_{4}}))$ then an appropriate pairwise kernel would be

(1) $$\begin{array}{c} {\hat{K}}_{P1}=K\left(x_{i_{1}},x_{i_{3}}\right)K\left(x_{i_{2}},x_{i_{4}}\right)+K\left(x_{i_{1}},x_{i_{4}}\right)K\left(x_{i_{2}},x_{i_{3}}\right). \end{array}$$

Subsequently, we will use the loose convention that the arguments of the pairwise kernel can be data vectors, **x**_*i*_, or derived kernel matrices, *K*(**x**_*i*_, **x**_*j*_). Ben-Hur and Noble [5] proposed kernel ${\hat{K}}_{P1}$ and called it the tensor product pairwise kernel (TPPK). This pairwise kernel can be viewed as the weighted adjacency matrix of a Kronecker product graph of two graphs associated with the constituent kernels [6].

The second pairwise kernel we consider is [7]

(2) $$\begin{array}{c} {\hat{K}}_{P2}=K\left(x_{i_{1}},x_{i_{3}}\right)+K\left(x_{i_{1}},x_{i_{4}}\right)+K\left(x_{i_{2}},x_{i_{3}}\right)+K\left(x_{i_{2}},x_{i_{4}}\right). \end{array}$$

Assuming *K*(**x**_*i*_, **x**_*j*_) is a positive semidefinite (PSD) kernel then the sum or the product of two such PSD kernels is also a PSD kernel, hence establishing ${\hat{K}}_{P1}$ and ${\hat{K}}_{P2}$ as allowable PSD kernels. Our third pairwise kernel is called the metric learning pairwise kernel (MLPK) [8]:

(3) $$\begin{array}{c} {\hat{K}}_{P3}={\left[K(x_{i_{1}},x_{i_{3}})-K(x_{i_{1}},x_{i_{4}})-K(x_{i_{2}},x_{i_{3}})+K\left(x_{i_{2}},x_{i_{4}}\right)\right]}^{2}. \end{array}$$

A kernel is a mapped inner product *K*(**x**_*i*_, **x**_*j*_) = Φ(**x**_*i*_)^*T*^Φ(**x**_*j*_); hence, ${\hat{K}}_{P3}$ follows from

(4) $$\begin{array}{c} {\hat{K}}_{P3}={\left[{\left(\Phi (x_{i_{1}})-\Phi (x_{i_{2}})\right)}^{T}\left(\Phi (x_{i_{3}})-\Phi (x_{i_{4}})\right)\right]}^{2}. \end{array}$$

Thus, for this kernel, the pair (**x**_*i*_1__, **x**_*i*_2__) is mapped to the vector Φ(**x**_*i*_1__) − Φ(**x**_*i*_2__) in feature space and the kernel is the inner product between these mapped vectors (subsequently squared). Extending this idea we can introduce a new kernel that is based on the inner product between the normalised pairs of vectors Φ(**x**_*i*_1__) − Φ(**x**_*i*_2__) and Φ(**x**_*i*_3__) − Φ(**x**_*i*_4__). This kernel is then based on the cosine similarity measure; that is,

(5) K^P4=Φxi1−Φxi2TΦxi1−Φxi2 ×Φxi1−Φxi2TΦxi3−Φxi4ooooo×Φxi3−Φxi4TΦxi3−Φxi4−1,

so

(6) K^P4=Kxi1,xi3−Kxi1,xi4−Kxi2,xi3+Kxi2,xi4 ×Kxi1,xi1−2Kxi1,xi2+Kxi2,xi2oooooo×Kxi3,xi3−2Kxi3,xi4+Kxi4,xi4−1.

For ${\hat{K}}_{P1}$, we mentioned the relation between this pairwise kernel and a Kronecker product graph. This motivates consideration of other types of product graphs and one based on a Cartesian product graph (CSPK) has been proposed by [6]. This kernel is defined by

(7) K^P5=Ki1,i3Ii2=i4+Ki2,i4Ii1=i3 +Ki1,i4Ii2=i3+Ki2,i3Ii1=i4,

where the (*i*, *j*)th component of a kernel matrix [*K*] quantifies the similarity between the *i*'th and *j*'th nodes and where *I*(*γ*) is an indicator function (1 if its argument is true and 0 otherwise). We include this kernel for completeness, since it will be included in our usage of MKL later. The information encapsulated in these product graphs can overlap substantially depending on the nature of the base kernels. The tensor product *G* × *G* and the Cartesian product *G* □ *G* of a graph *G*(*V*_*G*_, *E*_*G*_) use the same vertex set, defined as a Cartesian product over the vertices in *V*_*G*_ ({(*g*, *h*)∣*g*, *h* ∈ *V*_*G*_}). However, their edge sets are defined as follows [9]:

(8) EG×G=g,hg′,h′ ∣ gg′∈EG, hh′∈EG,EG □ G=g,hg′,h′ ∣ g=g′, hh′∈EG, or gg′∈EG, h=h′.

A base kernel with nonzero diagonal elements corresponds to a graph with self-edges (i.e., *gg* ∈ *E*_*G*_). In these cases a tensor product kernel will subsume a Cartesian product kernel over the same graph.

It is possible to further combine these types of pairwise kernels with other standard kernels, for example, Gaussian kernels or kernels based on polynomials; for example,

(9) $$\begin{array}{c} K={\left[K\left(x_{i_{1}},x_{i_{3}}\right)+K\left(x_{i_{2}},x_{i_{4}}\right)+r\right]}^{d}. \end{array}$$

However, these types of kernels also require the use and determination of a* kernel parameter*, for example, *r* in (9), via a further cross-validation study, and so we will not consider them further in this study. There are further non-PSD (infinite) symmetric pairwise kernels which have been considered [7]. Though it is possible to project these to the cone of positive semidefinite kernels and use a proxy kernel [10], we investigated these and did not find consistently good performance, so they are not considered further in this study.

To give equal weight to different types of data we can further normalize the base kernels. Thus, viewing the kernel as a mapped inner product [2], we used the mapping **x** → Φ(**x**)/‖Φ(**x**)‖_2_; then,

(10) K^xi,xj=Φ(xi)·Φ(xj)Φ(xi)·Φ(xi)Φ(xj)·Φ(xj)=Kxi,xjKxi,xiKxj,xj.

### 2.2. Multiple Kernel Learning

Different sources of data can be encoded into different types of* data kernel* [2], which we denote by *K*(**x**_*i*_1__, **x**_*i*_2__). Examples include diffusion kernels or standard kernels such as linear or Gaussian kernels [2] for encoding the similarity between data objects **x**_*i*_1__ and **x**_*i*_2__. These data kernels are, in turn, embedded in pairwise kernels, as described in the previous section. The resultant* pairwise kernels* will be denoted by ${\hat{K}}_{l}(x_{i},x_{j})$ (where ℓ = 1,…, *p*) and are the base kernels used to construct a* composite kernel*, denoted by $\bar{K}$, for MKL learning. Two distinct base kernels may be different pairwise kernels representing the same source of data (i.e., the same data kernel) or they could be the same type of pairwise kernel applied to two different sources of data.

With* multiple kernel learning* [3, 11, 12], we can derive a composite kernel, $\bar{K}$, as a linear combination of these base kernels:

(11) $$\begin{array}{c} \bar{K}\left(x_{i_{1}},x_{i_{2}},x_{i_{3}},x_{i_{4}}\right)=\overset{p}{\underset{l=1}{\sum }}\lambda_{l}{\hat{K}}_{l}\left(x_{i_{1}},x_{i_{2}},x_{i_{3}},x_{i_{4}}\right), \end{array}$$

where *λ*_ℓ_ are the* kernel weights* that are restricted to lie on the simplex:

(12) $$\begin{array}{c} \overset{p}{\underset{l=1}{\sum }}\lambda_{l}=1,\;\lambda_{l}\geq 0. \end{array}$$

The kernel weight *λ*_ℓ_ indicates the relative informativeness of data source ℓ. Aside from these weights, we must find the values of the learning parameters *α*_*i*,*j*_ during the training process. These learning parameters are the same learning parameters as for a standard Support Vector Machine [3]. However, in this case, rather than a single sample index, we use two indices, denoting the link between node *i* and *j*, since a data vector is attached to a link between two nodes and carries information about a possible interaction between these nodes. Here, we are interested in binary classification (link or nonlink) so *y*_*i*,*j*_ = ±1. Both *α*_*i*,*j*_ and *λ*_ℓ_ are found during the learning process through the following optimisation task:

(13) min⁡λ max⁡α  ∑i1,i2=1mαi1,i2   −12∑i1,…,i4=1mαi1,i2αi3,i4yi1,i2yi3,i4K¯xi1,xi2,xi3,xi4

subject to

(14) ∑i1,i2=1mαi1,i2yi1,i2=0, 0≤αi1,i2pppppp

and the constraints in (12). This optimisation problem for MKL [3] can be tackled via quadratically constrained linear programming [13] and other methods [11, 12]. If {*α*_*i*_1_,*i*_2__^⋆^, *λ*_ℓ_^⋆^} is the solution to the optimisation problem in (13), then the predicted class label for novel input data, **z**_*i*_, is given by the sign of

(15) $$\begin{array}{c} \phi \left(z_{i_{1}},z_{i_{2}}\right)=\overset{m}{\underset{j_{1},j_{2}=1}{\sum }}\alpha_{j_{1},j_{2}}^{⋆}y_{j_{1},j_{2}}\bar{K}\left(x_{j_{1}},x_{j_{2}},z_{i_{1}},z_{i_{2}}\right)+b^{⋆}, \end{array}$$

where

(16) b⋆=−12max⁡{i ∣ yi=−1}∑j=1mαj⋆yjK¯xi1,xi2,xi3,xi4 +min⁡{i ∣ yi=+1}∑j=1mαj⋆yjK¯xi1,xi2,xi3,xi4

which is an adapted version of the decision function and bias, *b*, of a Support Vector Machine [3], appropriate to the context presented here.

### 2.3. Introduction of a Probability Measure

In later experiments, we will introduce a confidence measure associated with linkage prediction. Most MKL methods have an intrinsic measure of confidence, namely, the margin measure *ϕ*(**z**) given in (15). The larger the absolute value of *ϕ*(**z**) the greater the degree of confidence in the predicted label. We can relate *ϕ*(**z**) to a probability measure by fitting a posterior probability distribution [14]. For binary classification, we use the sigmoid *p*(*y* = +1∣*ϕ*) = [1 + exp⁡(*Aϕ* + *B*)]^−1^. With binary labels for link *l*, *y*_*l*_ ∈ {−1,1}, we define *t*_*l*_ = 0.5(*y*_*l*_ + 1)∈{0,1}. The parameters *A* and *B* are then found by minimizing the negative log likelihood of the training data via the cross entropy error function:

(17) $$\begin{array}{c} \underset{A,B}{\min}\left[-\underset{l}{\sum }t_{l}\log\left(p_{l}\right)+\left(1-t_{l}\right)\log\left(1-p_{l}\right)\right], \end{array}$$

where *p*_*l*_ is the sigmoid probability function evaluated from *ϕ*(**z**) for the link considered. To minimize this function, we used the Levenberg-Marquardt algorithm [15].

## 3. Results

In this paper, we set out to investigate the following questions. Firstly, which pairwise kernel is the most accurate. As a second objective, we considered MKL and the gain to be made by using a weighted combination of different types of data over using a uniform combination. Combined with our first objective, a further objective was to understand if one type of pairwise kernel is the best or if higher accuracy is achieved by using a weighted combination of pairwise kernels. Our results are reported in Section 3.1. We then place a probability measure on *ϕ*(**z**_*i*_1__, **z**_*i*_2__) in (15) and briefly consider prediction restricted to high confidence inference (Section 3.2) and strategies for removing possibly wrongly labelled datapoints in the training data (Section 3.3).

### 3.1. Multiple Kernel Learning

For our analysis, we used kernels from six heterogeneous data sets that have been used for supervised interactive network inference in a previous study [1]: three based on protein sequence kernels and three based on diffusion kernels. Borrowing notation from these authors, we used three data kernels based on sets of amino acid sequences (spectrum (*K*_*S*_) [4], motif (*K*_*M*_) [16], and Pfam (*K*_*P*_)) [17] and three diffusion data kernels based on interaction networks from the BioGRID database [18] (yeast two-hybrid assay (*K*_YH_), genetic interactions (*K*_GI_), and affinity capture-MS (*K*_MS_)) [1].

In their original study, Qiu and Noble [1] used a uniformly weighted combination of kernels: the average value of the three sequence kernels was added to the average of the three diffusion kernels (we omit using their RBF kernels, given the latter contain a kernel parameter). A tensor product pairwise kernel (TPPK or *P*1 in our classification) was applied as follows:

(18) $$\begin{array}{c} \bar{K}={\hat{K}}_{P1}\left(\frac{K_{M}+K_{S}+K_{P}}{3}+\frac{K_{\text{YH}}+K_{\text{GI}}+K_{\text{MS}}}{3}\right). \end{array}$$

Here, we use MKL to assign weights according to the contribution of each data source for predicting edges in a gene interaction network. Since uniform weighting is a subinstance of using variable kernel weights, MKL will inevitably improve on (or equal) a uniform weighting scheme. The data we are using provides information on individual proteins, rather than protein pairs, and hence we use pairwise kernels, as outlined above. Since we have kernel weights *λ*_ℓ_ and sequence or diffusion kernels *K*_ℓ_, for a given pairwise kernel, ${\hat{K}}_{P}$, our composite kernel after MKL training will be

(19) $$\begin{array}{c} {\bar{K}}_{P}=\overset{p}{\underset{l=1}{\sum }}\lambda_{l}{\hat{K}}_{P}\left(K_{l}\right). \end{array}$$

We used the simple MKL Matlab package [19]. Training is compute-intensive, even with an efficient implementation, so we learned the kernel weights using relatively small sets of 1,000 to 4,000 examples. We found that the kernel weights for data sets larger than 4,000 examples were barely altered, so we did not use larger data sets for this purpose. The learnt weights for each individual pairwise kernel appear in Table 1. Of the three sequence data kernels, the Pfam kernel (*K*_*P*_) achieves the highest weight for the TPPK kernel ${\bar{K}}_{P1}$. By contrast, the motif kernel (*K*_*M*_) was assigned zero weight in all cases but ${\bar{K}}_{P2}$. There is a greater difference in the way these pairwise kernels apply information from the diffusion kernels. The TPPK (${\bar{K}}_{P1}$) and CSPK (${\bar{K}}_{P5}$) kernels rely almost entirely on the affinity capture-MS data, while the ${\bar{K}}_{P2}$ and ${\bar{K}}_{P4}$ kernels are able to leverage information from the yeast two-hybrid assay and gene interaction data as well. No pairwise kernel uses more than five of the component data kernels. The ${\bar{K}}_{P1}$ kernel weights exhibit the highest variation, while the ${\bar{K}}_{P4}$ kernel has a more even distribution of weights. Once the MKL algorithm had learned the weights, we recomputed the kernels as described in (19) and compared the kernels' performance.

The* S. cerevisiae* data from [1] form a balanced set consisting of 10,980 positive and 10,980 negative pairs of interacting genes (21,960 total pairs). Given this relatively large data set, we wished to see how well each kernel would perform when trained on subsets of different size. Thus, we ran three different experiments on these data. To assess performance on small data sets, we split the original set into 20 subsets of 1,098 examples each, randomly assigning an equal number of positive and negative examples to each subset. We ran 5-fold cross-validation to obtain average accuracy and AUC (area under the ROC curve) values for each kernel on each subset. Following the recommendations in [20] for comparing multiple classifiers on multiple data sets, we ranked the kernels for each data set and used nonparametric tests to assess differences between the kernels. We used the Friedman test to determine the significance of differences between all five kernels and then used the post hoc Nemenyi test to assess pairwise differences [12, 20]. To evaluate the kernels' performance on medium and large data sets, we used the same procedure, splitting the original data set into 10 subsets of 2,196 examples (1,757 training/439 test per fold) or 5 subsets of 4,392 examples (3,514 training/878 test per fold).

We expect this experimental design to yield realistic results for the data used in our study [21], but to extend this work to general-purpose classifiers, we recommend separating test data into separate classes as outlined in [22].

#### 3.1.1. Comparison of Different Pairwise Kernels

**Figure 1:**
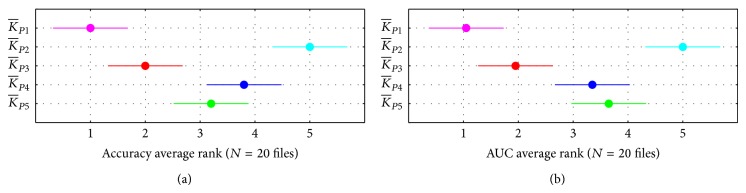
Comparison of average rankings for accuracy (a) and AUC (b) for 20 small data sets using unweighted pairwise kernels. The dot for each kernel identifies its mean rank; horizontal bars depict the Nemenyi test critical region for *α* = 0.05. The tensor product kernel (${\bar{K}}_{P1}$) consistently had the highest ranking while the symmetric direct sum kernel (${\bar{K}}_{P2}$) had the lowest. The differences between the remaining three kernels become clearer when we consider AUC as well as accuracy: the metric learning (${\bar{K}}_{P3}$) kernel has higher rankings than the other two on both measures.

For small data sets, the tensor product kernel (${\bar{K}}_{P1}$) consistently yields the highest accuracy ranking of any pairwise kernel (mean 1.0) while the symmetric direct sum kernel (${\bar{K}}_{P2}$) consistently yields the lowest (Figure 1). The metric learning (${\bar{K}}_{P3}$), cosine-like (${\bar{K}}_{P4}$), and Cartesian graph product (${\bar{K}}_{P5}$) pairwise kernels yield intermediate rankings, though the ${\bar{K}}_{P3}$ kernel (mean 2.0) was consistently ranked higher than the other two. When we rank the kernels based on AUC score as well as accuracy, we again see that the ${\bar{K}}_{P3}$ kernel yields higher performance than ${\bar{K}}_{P4}$ or ${\bar{K}}_{P5}$, but here the ${\bar{K}}_{P4}$ ranking is higher than that for ${\bar{K}}_{P5}$, making it difficult to identify a clear winner between them. The ${\bar{K}}_{P1}$ kernel's high accuracy and AUC rankings are statistically significant (*α* = 0.01) when compared to all but the ${\bar{K}}_{P3}$ kernels, but the differences between ${\bar{K}}_{P1}$ and ${\bar{K}}_{P3}$ are not statistically significant at *α* = 0.05. Results for medium and large data sets (not shown) are nearly identical, but the smaller data size yields less statistical power.

#### 3.1.2. Performance of Individual Pairwise Kernels with Multiple Types of Input Data

We compared the performance of each individual pairwise kernel with and without MKL weights using the same cross-validation procedure outlined above. To determine whether MKL yields significant improvements for any of the kernels, we use a Wilcoxon signed rank test for *N* = 10 and *N* = 20 files and a paired *t*-test for *N* = 5 data files (there are no critical values for the Wilcoxon test for *α* ≤ 0.05 and *N* = 5).  Table 2 shows the relative performance of the weighted and averaged kernels. In many cases we find a statistically significant increase in performance if we use weighted kernels (weighted over the 6 constituent kernels); even if the difference is not significant, it is rare that weighted kernels limit performance. In particular, the weighted version of the ${\bar{K}}_{P3}$ kernel exhibits significantly higher accuracy than the unweighted version in all of our experiments. On large training sets, we see a significant improvement with the weighted versions of the ${\bar{K}}_{P2}$, ${\bar{K}}_{P3}$, and ${\bar{K}}_{P4}$ kernels: increases in accuracy range from 2.2% to 3.6%. We note that the weighted version of the ${\bar{K}}_{P5}$ kernel yields slightly lower accuracy on average than the unweighted version, but these differences are not statistically significant.

**Figure 2:**
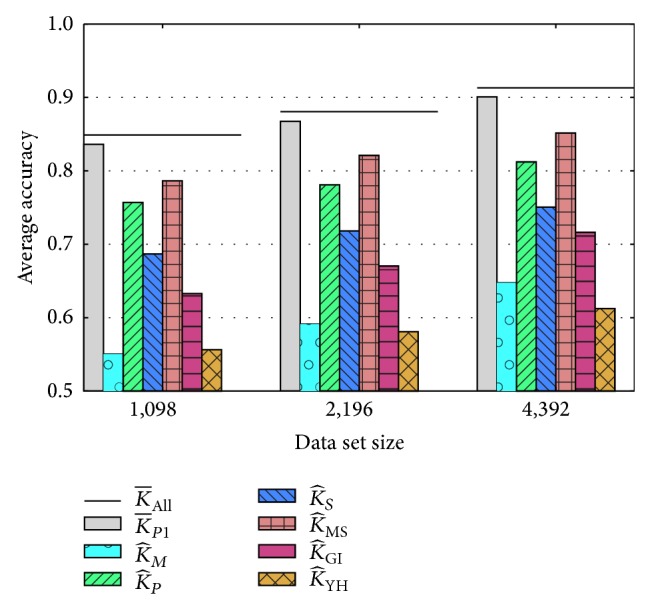
Graphical depiction showing the typical improvement in accuracy we see when using a weighted sum of base kernels via MKL. Here, we compare the average performance of the best-performing composite kernel, ${\bar{K}}_{P1}$ (solid grey bars), with the corresponding base kernels (hashed bars) on data sets of three different sizes. By leveraging information from multiple kernels, ${\bar{K}}_{P1}$ provides an accuracy increase of 4% to 5% over the best of the base kernels. When we use MKL over all 30 base kernels combined (${\bar{K}}_{\text{All}}$), we achieve a further 1.2% to 1.4% increase (black bars). Differences between ${\bar{K}}_{P1}$ and its base kernels are significant at *α* < 0.001; differences between ${\bar{K}}_{\text{All}}$ and ${\bar{K}}_{P1}$ are significant at *α* < 0.01.

Secondly, we compared the relative performance of these composite MKL kernels with their corresponding base kernels. We ran the same experiment outlined above on the individual base kernels. In general, we see a significant difference between the MKL-weighted kernels and their individual base kernels. For example, the top-performing combined kernel ${\bar{K}}_{P1}$ yields accuracy that is at least 4% higher than the nearest corresponding base kernel (Figure 2). We note that the weights used for the constituent kernels roughly track the relative performance of the kernels: for example, *K*_*P*_ and *K*_MS_ yield the highest accuracy and also have the largest weights for ${\bar{K}}_{P1}$ (see Table 1), while the two weakest base kernels, *K*_*M*_ and *K*_YH_, have zero weights and do not contribute to the final composite kernel.

#### 3.1.3. Performance Using All Pairwise Kernels and All Types of Input Data

Next we use MKL with all five pairwise kernels and all six different types of input data to produce a comprehensive kernel, ${\bar{K}}_{\text{All}}$. This gave 30 possible kernels but only 11 of these have nonzero kernel weights (Table 3). Notably, the tensor product kernel (${\bar{K}}_{P1}$) and the metric learning kernel (${\bar{K}}_{P3}$) contribute 4 and 3 base kernels, respectively. None of the motif base kernels (*K*_*M*_) are included, nor are any of the Cartesian product base kernels (${\bar{K}}_{P5}$). The resulting ${\bar{K}}_{\text{All}}$ kernel yields accuracy that is 1.2% to 1.4% higher than the best individual pairwise kernel (horizontal lines in Figure 2). For all data set sizes tested, this difference is statistically significant. The kernel weights and the improved performance both indicate that there is complimentary information provided by the different pairwise kernels. By contrast, the closely related Cartesian product kernels and tensor product kernels likely yield redundant information (Section 2.1), resulting in zero weights for Cartesian product base kernels.

### 3.2. Cautious Classification

We now introduce the probability measure considered in Section 2.3. A confidence measure is of interest in its own right. However, our interest here is in its use to further improve test accuracy for the pairwise-kernel based MKL scheme already introduced. Specifically, we consider* cautious classification* in which we decline to make predictions if the confidence is sufficiently low but make predictions of a link or nonlink in high confidence instances. For the* S. cerevisiae* data set, we show that this strategy can yield significant improvements in test accuracy, though at the cost of a reduced set of predictions.

**Figure 3:**
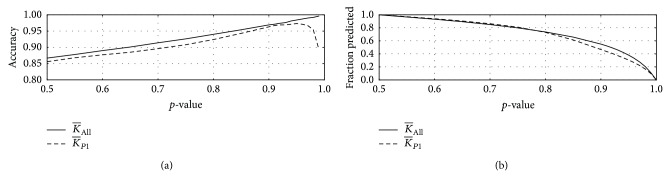
Plot of the test accuracy ((a) *y*-axis) and fraction of pairs predicted ((b) *y*-axis) as a function of the *p*-value cutoff (*x*-axis) for (i) using all available pairwise and data kernels (${\bar{K}}_{\text{All}}$, solid curve) and (ii) the top-performing pairwise kernel (${\bar{K}}_{P1}$, dashed curve). By increasing the *p*-value cutoff, we increase the accuracy in our predictions but decrease the fraction of pairs for which we can make predictions.

In Figure 3 we plot the test accuracy (as a fraction) versus the *p*-value cutoff (a) when using all the above mentioned pairwise and data kernels. The test accuracy increased up to 0.996 as we increased the *p*-value cutoff, while the number of points predicted dropped to 246 (11.2%). If we used individual pairwise kernels with all the available data (we illustrate with ${\bar{K}}_{P1}$ in this figure), then the test accuracy was lower (0.86 to 0.97 for ${\bar{K}}_{P1}$), but, as illustrated, we also noticed a greater sensitivity to outliers (incorrect link-labels) for high values of the *p*-value cutoff. These numerical simulations are for *m* = 2,196 and so they correspond to the weighted values for *N* = 10 in Table 2 when the cutoff is *p* = 0.50.

### 3.3. Data Cleaning

To address the impact of outliers on our classifiers, we investigated two data cleaning methods. In each method, our goal was to train an SVM using as many informative examples as possible while eliminating counter-productive examples (outliers). In both cases, we initiated training with a small subset of reliably labelled datapoints, where the* label* of link (positive) or nonlink (negative) is known. To obtain reliable representatives from both positive and negative example classes, we estimated the centroids of each class and chose the 10 datapoints in each class that were closest to their centroids (alternatively, biological insight may give a reliable starting set). We then learnt the remaining datapoints sequentially and avoided potential outliers using one of two strategies. Our first approach, introduced by [3], is to predict the labels for all currently unlearnt links in the training data and use the datapoint with the lowest associated confidence for training in the next iteration. This procedure tends to postpone learning potential outliers to the end of the learning process but incurs a high computational cost as it makes predictions for all unlearnt links at each iteration. A second and less computationally costly approach is to select the next training example randomly at each iteration and predict its label using the current classifier. If the prediction is high confidence but the actual label is of opposite sign, we omit the datapoint since it may be an outlier.

**Figure 4:**
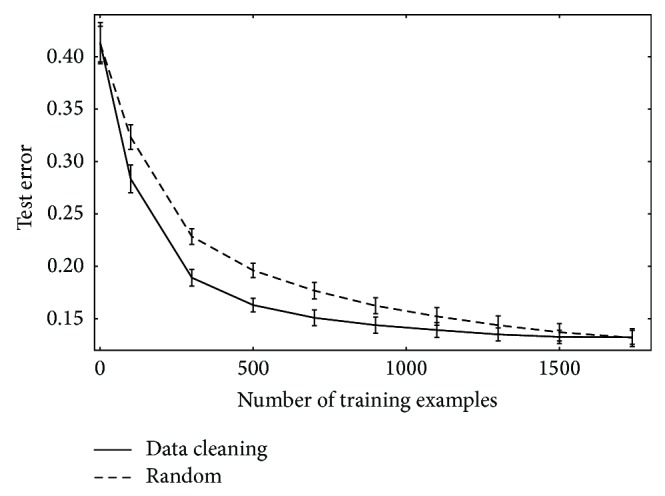
Mean test error as a fraction (*y*-axis) versus the number of patterns learnt (*x*-axis) for the top-performing pairwise kernel, ${\bar{K}}_{P1}$. Error bars depict a 95% confidence interval for 5-fold cross-validation test error averaged over 10 distinct data subsets, each with *m* = 2,196. The upper curve gives the performance if we learn all the data sequentially (from a common start set) in random order. The lower curve gives the test accuracy if the next addition to the training set is chosen based on having the lowest confidence predicted link-label.

For the data set considered [1], there appear to be few anomalous links in the data, so there is at most a small gain in test accuracy when we use these methods. In Figure 4, we give the test error achieved on held-out data, averaged over 10 distinct data sets from the experiments described in Section 3.1. In this case, we are making predictions of link-labels over all currently unlearnt datapoints and learning that datapoint with the lowest associated confidence for the link-label. The learning curve has a shallow minimum of the test error with a fractional test error of 0.1380 at *m* = 1563, against a final test error of 0.1490 at *m* = 2000, having learnt all the data in the training set. Of course, we can also lessen the influence of outliers by using an *L*_1_ or *L*_2_ soft margin with a margin-based classifier [2, 3]. However, when using a soft margin, we need to pursue a validation study, using some held-out data, to establish the most appropriate value for the soft margin parameter. With the proposed data cleaning method, there is no need to use validation data since there is a suitable stopping criterion available. Specifically, we can stop learning new datapoints when the equivalent of the margin band is empty [3], that is, when |*ϕ*(**x**_*i*_1__, **x**_*i*_2__)| > 1 in (15). At this point, we would be learning two types of link-labels. Either we learn a link-label of the expected sign, that is, the predicted link-label and actual label agree, or the predicted link-label and actual label disagree. If the predicted and actual link-labels agree then this potential link is the equivalent of a non-support vector, with *α*_*i*_1_,*i*_2__^⋆^ = 0, and so it will not contribute to the decision function stated in (15). We therefore do not need to learn this datapoint. Alternatively, the new link will have a label that is substantially out-of-alignment with the current hypothesis (after having learnt a number of link-labels). With |*ϕ*(**x**_*i*_1__, **x**_*i*_2__)| > 1, it is being placed within the data space of the oppositely labelled datapoints. Such a link could be correct, but it does have a strong possibility of being an outlier. We would not stop before the margin band is empty because the newly learnt datapoints will have *α*_*i*_1_,*i*_2__^⋆^ > 0 and thus will contribute to the decision criterion stated in (15). This stopping criterion gave a termination point that is within 0.1% of the empirically observed minimum error, with cessation of learning after 1,642 samples, with a test error of 0.1323, as against the observed minimum test error of 0.1319 at 1,565 samples learnt. Beyond this stopping point, the test error can rise as we may start learning links (or nonlinks) which are anomalously labeled.

An additional advantage of using this sequential learning method is that the prospects of achieving convergence with a linear kernel are enhanced. Specifically, a mislabelled datapoint can appear as a wrongly labelled datapoint within a cluster of datapoints of the opposite sign. This would mean the two classes of data can become nonseparable, requiring the use of a nonlinear kernel (e.g., an RBF kernel), with an associated validation study to find the appropriate value of the kernel parameter.

## 4. Conclusion

In this paper, we have investigated supervised interactive network inference using multiple kernel learning. Our objective was to consider ways to improve prediction performance and there are five main conclusions drawn from our study. Firstly, we compared five different types of pairwise kernel, which did not require adjustment of a kernel parameter, on six different types of data for supervised network inference. Our conclusion was that the pairwise kernel *P*1 (TPPK) worked best. Next, we considered whether use of a weighted combination of kernels (data sources) performed better than a uniformly weighted combination (Table 2) and, as expected, we found this was the case. Thirdly, for each pairwise kernel, we established performance using MKL over these six different data kernels and then compared this with the performance of MKL, when using all five different types of pairwise kernel and taken over all six different types of data; that is, the algorithm could use a weighted combination of 30 different types of kernel. At a statistically significant level, we found that this 30-base kernel combination outperformed the best of the individual pairwise kernels taken in isolation by between 1.2 and 1.4 percentage points. Thus, TPPK may look like the most effective pairwise kernel, but there must be complementary information among these different types of pairwise kernels and they are best used in combination with kernel-selection being made by the algorithm. To further improve predictive test accuracy, we next introduced a confidence measure associated with the class assignment. We showed that there are significant gains from using cautious classification, where prediction is confined to a high confidence instance. Our fifth study was to investigate the use of this probability measure with data cleaning. The* S. cerevisiae* data set considered appears clean, with only a few link-labels suggested as being possibly mislabelings. Thus, this strategy only gave a gain of 1.7% in our study in Section 3.3. However, label noise may be a more substantial problem in the understanding of pathways in more advanced organisms. This strategy would therefore likely yield better gains in these contexts.

In short, each component strategy has delivered modest through to more substantive improvements in predictive accuracy. Taken together, though, they lead to a substantial improvement in predictive accuracy over previous studies [1] and a highly accurate predictor.

As a consequence of this investigation, we have identified several potentially fruitful avenues for future work. We selected the SimpleMKL method for its speed and relatively sparse kernel weights, but other weighting methods conceivably could provide better performance [12, 23]. Further, recently proposed methods for predicting protein interactions such as coevolutionary divergence [24] and remote homology [25] could be used to extend our model. Finally, we have enumerated several approaches to data cleaning that could become increasingly effective as novel data sets become available.

## Figures and Tables

**Table 1:** Kernel weights for the pairwise kernels used in this study. The weights selected for each kernel were those at the highest *C*-value that had two or more nonzero weights.

Kernel	Kernel weights for individual models					
	${\hat{K}}_{M}$	${\hat{K}}_{P}$	${\hat{K}}_{S}$	${\hat{K}}_{\text{GI}}$	${\hat{K}}_{\text{YH}}$	${\hat{K}}_{\text{MS}}$
${\bar{K}}_{P1}$	0	0.449	0.099	0.033	0	0.419
${\bar{K}}_{P2}$	0.362	0.198	0	0.096	0.308	0.035
${\bar{K}}_{P3}$	0	0.258	0	0.200	0	0.542
${\bar{K}}_{P4}$	0	0.170	0.176	0.211	0.191	0.252
${\bar{K}}_{P5}$	0	0.266	0	0	0	0.734

**Table 2:** Cross-validation results for the pairwise kernels using unweighted (U) and weighted (W) combinations of the six unpaired kernels for data sets of different sizes. Shown is test accuracy averaged over *N* = 20, *N* = 10, or *N* = 5 data sets (1,098, 2,196, or 4,392 examples, respectively, split into 80% training and 20% test sets). In many cases, the MKL weights yield a significant improvement while in other cases there is no significant change. Significant values are denoted as follows: ^**^Wilcoxon signed rank *α* = 0.01 or ^*^ *α* = 0.05, and ^†^paired *t*-test *α* < 0.01. Statistically significant values are marked in bold type.

Kernel	*N* = 20		*N* = 10		*N* = 5	
	U	W	U	W	U	W
${\bar{K}}_{P1}$	0.826	0.836^*^	0.860	0.867	0.895	0.901
${\bar{K}}_{P2}$	0.667	0.662	0.663	0.681^**^	0.694	0.716^†^
${\bar{K}}_{P3}$	0.764	0.801^**^	0.802	0.837^**^	0.852	0.883^†^
${\bar{K}}_{P4}$	0.731	0.740	0.756	0.764	0.755	0.791^†^
${\bar{K}}_{P5}$	0.764	0.759	0.817	0.807	0.862	0.849

**Table 3:** Kernel weights learned for a comprehensive kernel, ${\bar{K}}_{\text{All}}$, that combines all base pairwise kernels. For each pairwise kernel, we show the final weight assigned to each of its base kernels. The tensor product kernel (${\bar{K}}_{P1}$) and the metric learning kernel (${\bar{K}}_{P3}$) contribute the most information to this comprehensive kernel. None of the motif base kernels (*K*_*M*_) contribute, nor do any of the Cartesian product base kernels (${\bar{K}}_{P5}$). The kernel weights sum to unity.

Kernel	Kernel weights for combined model					
	*K*_*M*_	*K*_*P*_	*K*_*S*_	*K*_GI_	*K*_YH_	*K*_MS_
${\bar{K}}_{P1}$	0	0.193	0	0.103	0.075	0.372
${\bar{K}}_{P2}$	0	0.002	0	0.010	0	0
${\bar{K}}_{P3}$	0	0.044	0	0.023	0	0.153
${\bar{K}}_{P4}$	0	0.006	0	0.019	0	0
${\bar{K}}_{P5}$	0	0	0	0	0	0
